# Engineering the Gut Microbiome: Emerging Genome-Editing Strategies and Therapeutic Applications

**DOI:** 10.3390/microorganisms14061174

**Published:** 2026-05-22

**Authors:** Liu Wu, Zongyan Li, Jinxuan Zhu, Zhigang Sun, Lujun Yan, Mingzhan Luo, Huahai Chen, Yeshi Yin

**Affiliations:** 1 College of Animal Science and Technology, Guangxi University, Nanning 530004, China; wuwillow5656@163.com (L.W.); ashtonjk@163.com (Z.L.); zjx512000@163.com (J.Z.); sl612429@163.com (Z.S.); lujunyan1227@163.com (L.Y.); 18568798058@163.com (M.L.); 2 Guangxi Academy of Marine Sciences, Guangxi Academy of Sciences, Nanning 530007, China; 3 Guangxi Key Laboratory of Animal Reproduction, Breeding and Disease Control, Nanning 530004, China; 4 Guangxi Zhuang Autonomous Region Engineering Research Center of Veterinary Biologics, Nanning 530004, China

**Keywords:** gut microbiome, genome editing technologies, delivery systems, targeted design strategies

## Abstract

The gut microbiome, often termed the human “second genome”, profoundly influences host physiology through metabolic interactions, immune modulation, and gut–brain axis signaling. Dysbiosis is implicated in the pathogenesis of obesity, inflammatory bowel disease (IBD), malignancies, and neuropsychiatric disorders. However, traditional gut microbiota interventions, such as probiotic supplementation and fecal microbiota transplantation (FMT), still exhibit significant limitations in precision therapeutics. Probiotic intervention fails to achieve precise regulation at the strain or genetic level, and although FMT demonstrates definitive efficacy against recurrent *Clostridioides difficile* infection (rCDI), its therapeutic outcomes and safety profiles show marked interindividual variability in ulcerative colitis (UC), metabolic syndrome, and other diseases, with insufficient treatment specificity to meet the practical demands of clinical precision intervention. Recent advancements in genome editing technologies, particularly Clustered Regularly Interspaced Short Palindromic Repeats (CRISPR)–CRISPR-associated (Cas) proteins systems and base editors, have enabled targeted functional manipulation of specific gut commensals and optimization of community architectures. These engineered strategies, combined with sophisticated delivery systems, demonstrate substantial potential in disease treatment, diagnostic monitoring, and immune modulation. This review systematically examines core editing methodologies, innovative delivery platforms, and targeted design strategies, elucidating their applications in metabolic disorders, IBD, cancer immunotherapy, and neuropsychiatric conditions. We critically analyze current technical bottlenecks and biosafety concerns while prospecting future directions, including in situ editing, artificial intelligence (AI)-driven design, and personalized engineering. Collectively, these insights aim to facilitate the clinical translation of gut microbiome engineering from bench to bedside.

## 1. Introduction

### 1.1. Physiological Roles of the Intestinal Microbiota

The gut microbiota constitutes a complex microbial ecosystem colonizing the host gastrointestinal tract, with bacteria as the predominant taxa alongside archaea, fungi, and bacteriophage-dominated viral communities [1,2]. At the phylum level, *Firmicutes* and *Bacteroidetes* represent the core dominant groups; however, their relative abundances exhibit substantial interindividual variability, ranging from 11% to 95% for *Firmicutes* and 0.6% to 86.6% for *Bacteroidetes*. Because phyla such as *Actinobacteria*, *Proteobacteria* (formally reclassified as *Pseudomonadota*), and *Verrucomicrobia* also contribute substantially to the overall community abundance, the combined relative abundance of the two dominant phyla is often below 90% [3]. Furthermore, the *Firmicutes*/*Bacteroidetes* ratio demonstrates highly dynamic characteristics modulated by dietary patterns, geographic location, and sequencing methodologies [3,4]. The gut microbiota establishes intimate symbiotic relationships with the host, maintaining physiological homeostasis through three principal pathways: metabolic interactions, immune modulation, and neural regulation.

In terms of metabolic interactions, the gut microbiota ferments dietary fibers to generate short-chain fatty acids (SCFAs), primarily acetate, propionate, and butyrate, which are produced in the human colon at an approximate concentration ratio of 60:20:20 (mM/kg) [5,6]. This ratio is profoundly modulated by dietary substrate composition and structure: fermentable polysaccharides serve as preferred carbon sources that promote the proliferation and metabolic activity of butyrate-producing bacteria (e.g., *Faecalibacterium prausnitzii* and *Roseburia* spp.) via glycolytic pathways; conversely, excessive protein intake shifts microbial metabolism toward proteolysis, generating branched-chain fatty acids, ammonia, and hydrogen sulfide (H_2_S), which competitively suppress butyrate synthesis [7]. Among these metabolites, butyrate serves as the primary energy source for colonic epithelial cells (colonocytes) and exerts pleiotropic effects through two distinct yet complementary mechanisms: potent inhibition of histone deacetylase (HDAC) activity to regulate gene transcription, and differential activation of G protein-coupled receptors. Among SCFA receptors, G protein-coupled receptor 43 (GPR43) can be activated by acetate, propionate, and butyrate, whereas G protein-coupled receptor 41 (GPR41) primarily responds to propionate and butyrate, exhibiting extremely low affinity for acetate [6]. Of particular importance within the SCFA family, butyrate is the principal endogenous ligand for GPR109A, a receptor that displays significantly higher affinity for butyrate than for acetate and propionate [6,8]. The signaling network jointly mediated by HDAC inhibition and GPR109A activation positions butyrate as a critical regulator in maintaining intestinal immune tolerance and barrier homeostasis [8]. In contrast, acetate primarily signals via GPR43, while propionate and butyrate act through both GPR41 and GPR43, collectively modulating host glucose and lipid metabolism and insulin sensitivity [6,9]. Furthermore, upon activation of GPR41/GPR43 on intestinal epithelial cells, SCFAs trigger the extracellular signal-regulated kinase 1/2 (ERK1/2) and p38 mitogen-activated protein kinase (MAPK) signaling cascades, rapidly inducing the production of chemokines and cytokines in cultured cells and mouse models [10]. These processes are essential for leukocyte recruitment and effector T cell activation in the intestine, thereby mediating protective immune responses and tissue inflammation in mice [10]. Additionally, the gut microbiota participates in the biosynthesis of essential micronutrients, including vitamin K and B-complex vitamins [11,12], and directly mediates the metabolism and biotransformation of xenobiotic compounds [13,14].

In terms of immune modulation, the gut microbiota engages in dynamic interplay with the intestinal mucosal immune system to sustain immune homeostasis [15]. Exopolysaccharides (EPS) secreted by specific *Bifidobacterium* strains modulate dendritic cells via the Toll-like receptor 2 (TLR2) signaling pathway, promoting regulatory T cell (Treg) differentiation and thereby suppressing the release of pro-inflammatory cytokines such as interleukin-6 (IL-6) and tumor necrosis factor-α (TNF-α) [16]. However, this equilibrium is inherently fragile: dysbiosis, exemplified by the overexpansion of *Pseudomonadota*, compromises intestinal barrier integrity, enabling bacterial products (e.g., lipopolysaccharide [LPS]) to translocate into the bloodstream and activate pattern recognition receptors, including Toll-like receptor 4 (TLR4), which precipitates local and systemic immune dysregulation [17,18].

Neurally, the gut microbiota regulates central nervous system (CNS) function along the microbiota–gut–brain (MGB) axis. SCFAs can cross the blood–brain barrier (BBB) via monocarboxylate transporters, yet their physiological circulating concentrations are low, and direct BBB permeation remains controversial. Thus, peripheral GPR41/FFAR3 and GPR43/FFAR2 signaling, immune modulation, and vagal afferent pathways are regarded as the major routes for microbiota–CNS crosstalk [19,20,21]. Tryptophan metabolites (kynurenine and indole derivatives) further modulate neuroimmunoendocrine signaling through the aryl hydrocarbon receptor [20].

The vagus nerve constitutes the core anatomical pathway for bidirectional gut–brain communication [22,23]. Vagal afferents convey intestinal interoceptive and microbial signals to the brainstem nucleus tractus solitarius, whereas parasympathetic efferents control gastrointestinal motility and the cholinergic anti-inflammatory reflex [23]. Microbial metabolites detected by vagal terminals remodel central autonomic and mood-related circuits, while vagal efferent activity restrains peripheral inflammation by inhibiting macrophage pro-inflammatory cytokines such as TNF [23]. For instance, *Lactobacillus rhamnosus* JB-1 modulates brain gamma-aminobutyric acid (GABA) receptor expression and mitigates anxiety-like behaviors in a vagus-dependent fashion, and these effects are eliminated by subdiaphragmatic vagotomy [24].

Gut commensals also produce and regulate major neurotransmitters, including GABA, dopamine, 5-hydroxytryptamine (5-HT), and acetylcholine [24,25,26]. Under physiological conditions, GABA penetrates the BBB via specific transporters, whereas serotonin and dopamine show poor BBB permeability [24,25]. Accordingly, microbiota-derived neurotransmitters exert neuromodulatory effects mainly through peripheral pathways, including enteric nervous system regulation, vagal afferent signaling, and host neurotransmitter metabolism, whereas their direct central actions remain incompletely clarified [24,25,26,27,28,29].

However, dysbiosis has been increasingly implicated in causal relationships with various disease states. In metabolic disorders, obesity is associated with altered gut microbiota composition; notably, the directionality of change in the *Firmicutes*/*Bacteroidetes* ratio remains a subject of considerable debate, with studies reporting inconsistent findings regarding its increase or decrease in obese individuals [3,30,31]. Although fecal SCFA levels are frequently elevated in obese individuals, the abundance of specific butyrate-producing taxa, such as *Faecalibacterium prausnitzii*, may be diminished, suggesting that shifts in microbial metabolic function do not necessarily parallel taxonomic alterations [3,30]. In IBD, encompassing UC and Crohn’s disease (CD), a marked depletion of beneficial commensals, particularly *Faecalibacterium prausnitzii*, has been consistently observed and is associated with disease activity and postoperative recurrence [32,33,34]. In the oncological context, the gut microbiota modulates cancer pathogenesis and therapeutic responses through regulation of the tumor microenvironment; for instance, *Fusobacterium nucleatum* is enriched in colorectal cancer tissues, where it promotes tumor progression, metastasis, and induces chemoresistance through multiple molecular pathways, including TLR4/NF-κB activation, autophagy induction, and immunosuppressive microenvironment construction [35,36,37,38]. Regarding neuropsychiatric conditions, individuals with autism spectrum disorder (ASD) exhibit reduced gut microbiota diversity and dysregulated neurotransmitter metabolism, characterized by altered GABA/glutamate ratios and impaired serotonergic signaling, which may exacerbate social deficits via the MGB axis [39,40,41].

### 1.2. Limitations of Conventional Microbiome Intervention Strategies

Conventional microbiome modulation strategies harbor inherent limitations in precision and colonization stability, rendering them inadequate for personalized therapeutic applications. Current probiotic formulations predominantly derive from *Lactobacillus* and *Bifidobacterium* genera and aim to improve microbial community structure through exogenous supplementation of beneficial strains. However, most strains exhibit transient colonization patterns, with intestinal residence times varying considerably based on strain-specific traits and host background, typically resulting in clearance within days to weeks following discontinuation of administration [42,43,44,45]. Furthermore, the lack of target specificity in strain-mediated biological effects contributes to substantial interindividual heterogeneity in therapeutic outcomes [43,46,47]. Humans display marked heterogeneity in genetic background, gut microbiome configuration, and physiological responses, leading to highly variable and often conflicting clinical effects of the same probiotic intervention across individuals [47]. This individual-level variability is further compounded by the strain-specific and dose-dependent nature of probiotic mechanisms, coupled with the absence of precision-tailored selection criteria for matching specific strains to host contexts [46]. Consequently, the clinical efficacy of probiotics remains inconsistent across trials, with high levels of methodological and biological heterogeneity substantially reducing the certainty of evidence [48]. Collectively, these limitations underscore the urgent need to transition from empirical, “one-size-fits-all” probiotic supplementation toward precision-tailored microbiome interventions that account for individualized host–microbe contexts [47].

Prebiotics, though capable of promoting the proliferation of specific beneficial taxa such as *Bifidobacterium* [49,50], frequently induce gastrointestinal intolerance symptoms, including bloating, flatulence, and abdominal discomfort [49,51,52]. In patients with severe baseline dysbiosis, therapeutic efficacy is substantially constrained by the paucity of functional recipient strains capable of metabolic cross-feeding, since the production of beneficial metabolites such as SCFAs relies on cooperative interactions between primary and secondary fermenters within a sufficiently diverse microbial community [53,54]. By contrast, FMT achieves clinical remission rates of 85–95% in rCDI by reconstructing intestinal microecology and restoring colonization resistance [54,55,56]. Nevertheless, this intervention confronts multiple unresolved challenges: donor stool may harbor pathogenic microorganisms, including viruses, parasites, and antibiotic resistance genes, that evade routine screening, thereby posing significant biosafety risks, particularly in immunocompromised recipients [56,57]; substantial inter-donor heterogeneity in microbiota composition and functional capacity contributes to highly variable therapeutic responses [54,55,58]; and the persistent absence of standardized protocols for sample collection, processing, preservation, and administration compromises treatment reproducibility and scalability [54,55,56].

Furthermore, broad-spectrum antibiotics, while eliminating pathogenic bacteria, indiscriminately disrupt the architecture of the gut commensal microbiota, reduce microbial diversity, weaken host colonization resistance, and consequently increase the risk of colonization by drug-resistant pathogens and secondary infections [59,60]. Such intervention is essentially a nonspecific perturbation of the gut microecology rather than precise targeted therapy. Narrow-spectrum antimicrobial agents, although improving selectivity to some extent, remain confined within the traditional framework of passively imposing pharmacological selective pressure. For instance, fidaxomicin demonstrates cure rates for *Clostridioides difficile* infection comparable to those of vancomycin, with even lower recurrence rates [61], and the bacteriocin thuricin CD exhibits potent antimicrobial activity against *C. difficile* while minimally disturbing beneficial gut commensals [62]. However, direct comparison in the context of the gut microbiota environment indicates that only thuricin CD possesses truly narrow-spectrum antimicrobial properties [63]. Thus, even the optimal narrow-spectrum agents can only act passively upon the existing microbiota without actively reprogramming microbial functions. Moreover, traditional approaches such as probiotic supplementation, prebiotic modulation, and FMT all represent macroscopic-level microbiota interventions. Constrained by issues including transient colonization, functional dependence, and safety concerns, these methods cannot precisely rewrite the functional genes of specific bacterial species. This critical gap underscores the urgent imperative for developing next-generation technologies through programmable, sequence-specific intervention modalities, decoupling pathogen clearance from commensal protection, thereby completing the strategic transition from passive selection to active editing. Genome editing technologies serve as the core pathway to achieve this paradigm shift.

### 1.3. Potential of Genome Editing Technologies

The advent of genome editing technologies has overcome the constraints inherent in conventional intervention strategies, providing unprecedented opportunities for precision engineering of the gut microbiome. Through the design of specific guide RNAs (gRNAs) or editing templates, these technologies enable targeted modification of functional genes in specific intestinal bacteria, including virulence factor ablation [64,65], enhancement of beneficial metabolite synthesis [66,67], or selective modulation of community structure via pathogen elimination or targeted knockdown of dominant taxa [68,69], thereby effecting a strategic transition from broad-spectrum intervention to targeted therapeutics. The BACTRINS system illustrates this capability, achieving in situ knockout of the *Stx2B* gene in Shiga toxin-producing *Escherichia coli* through conjugative delivery of CRISPR-associated transposase (CAST) [65]. This approach precisely inactivates the toxin gene without killing the pathogen or disrupting resident commensal communities, conceptually converting the toxigenic strain into a non-pathogenic colonizer. In a murine gut colonization model, BACTRINS demonstrated efficient conjugative delivery (reaching near 100% in the asymptomatic model) and high-efficiency genomic integration (80–90%) of the therapeutic payload into target Stx loci, with accelerated pathogen clearance observed in the asymptomatic infection model; however, statistically significant survival improvement was not achieved in the severe symptomatic model, where rapid disease kinetics and incomplete target coverage limited therapeutic efficacy, underscoring its current proof-of-concept status [65]. Concurrently, genome editing technologies are driving a paradigm shift in microbiome research from descriptive correlation analyses to causal mechanistic investigations. Unlike metagenomic sequencing-based association studies, genetic manipulation coupled with phenotypic validation enables direct establishment of causal roles for specific bacterial taxa or functional genes. For instance, the ex vivo genetically engineered butyrate-producing *Bacillus subtilis* strain BsS-RS06551, when administered to mice, significantly attenuated high-fat-diet-induced weight gain and improved insulin resistance, providing actionable molecular targets for precision treatment of metabolic disorders [67].

**Figure 1:**
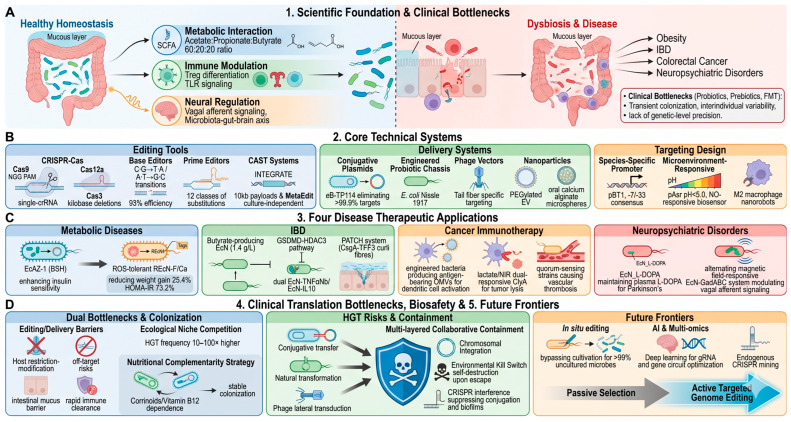
Schematic paradigm of gut microbiome genome editing for targeted disease therapy. (**A**) Scientific foundation and clinical bottlenecks: the intestinal microbiome maintains homeostasis via metabolic interactions (SCFA acetate:propionate:butyrate at a 60:20:20 ratio), immune modulation (Treg differentiation, TLR signaling), and neural regulation (vagal afferent signaling, microbiota–gut–brain axis), whereas dysbiosis drives metabolic disorders, IBD, colorectal cancer, and neuropsychiatric disorders; conventional interventions (probiotics, prebiotics, FMT) face limitations of transient colonization, interindividual variability, and lack of genetic-level precision. (**B**) Core technical systems: editing tools encompass CRISPR-Cas (Cas9, Cas12a, Cas3 for kilobase deletions), base editors (C·G → T·A and A·T → G·C transitions, 93% efficiency), prime editors (12 classes of substitutions, 10 kb payloads, MetaEdit, culture-independent), and CAST transposases (INTEGRATE); delivery systems include conjugative plasmids (eB-TP114 eliminating > 99.9% targets), engineered probiotic chassis (*E. coli* Nissle 1917), phage vectors with tail-fiber-specific targeting, and PEGylated oral calcium alginate microspheres; targeting design employs species-specific promoters (e.g., p*BT1*, −7/−33 consensus) and microenvironment-responsive circuits (p*Asr* pH ≤ 5.0, NO-responsive biosensors, M2 macrophage nanorobots). (**C**) Four disease therapeutic applications: for metabolic diseases, EcAZ-1 bile salt hydrolase (BSH) enhances insulin sensitivity and reactive oxygen species (ROS)-tolerant REcN-F/Ca reduces weight gain by 25.4% and homeostatic model assessment for insulin resistance (HOMA-IR) by 73.2%; for IBD, butyrate-producing *Escherichia coli* Nissle 1917 (EcN) (1.4 g/L), GSDMD–HDAC3 pathway modulation, the PATCH system with CsAgA–TFF3 curli fibres, and dual EcN-TNFαNb/EcN-IL10 systems are applied; for cancer immunotherapy, engineered bacteria produce antigen-bearing outer membrane vesicles (OMVs) to activate dendritic cells, lactate/NIR dual-responsive cytolysin A (ClyA) mediates tumor lysis, and quorum-sensing strains induce vascular thrombosis; for neuropsychiatric disorders, EcN L-DOPA maintains plasma L-DOPA levels in Parkinson’s disease, and an alternating magnetic field-responsive EcN-GadABC system modulates vagal afferent signaling via the microbiota-gut-brain (MGB) axis. (**D**) Clinical translation bottlenecks, biosafety, and future frontiers: editing and delivery barriers include host restriction–modification systems, off-target risks, intestinal mucus and immune clearance, and ecological niche competition with elevated HGT frequency; nutritional complementarity strategies (corrinoids/vitamin B12 dependence) enable stable colonization; multi-layered collaborative containment addresses HGT risks through chromosomal integration, environmental kill switches for self-destruction upon escape, and CRISPR interference (CRISPRi) suppressing conjugation and biofilms; future directions emphasize in situ editing to bypass cultivation for > 99% uncultured microbes, AI-driven multi-omics design via deep learning for gRNA and gene circuit optimization, endogenous CRISPR mining, and individualized precision medicine, transitioning from passive selection to active targeted genome editing. Created with Google Gemini (2026). Available at: https://gemini.google.com/app/dcbd3fd8b258f59c?hl=zh (accessed on 16 May 2026).

In summary, as illustrated in Figure 1, gut microbiome engineering, through the integration of genome editing technologies with advanced delivery systems, is transitioning from proof-of-concept validation toward clinical translation. This emerging discipline represents a pivotal direction in next-generation precision medicine, with substantial promise for applications in disease therapeutics, diagnostic monitoring, and health management.

## 2. Core Technologies of Gut Microbiome Genome Editing

The implementation of gut microbiome genome editing relies on the coordinated optimization of editing tools, delivery systems, and targeting design. Among these components, editing tools determine the efficiency and type of genetic modification. Delivery systems ensure the accurate access of these tools to targeted intestinal bacteria. Targeting design further enables the spatial and temporal control of the entire editing process. This section systematically reviews the research advances and application characteristics of these three categories of core technologies.

### 2.1. Development and Optimization of Editing Tools

Gut microorganisms exhibit substantial heterogeneity in oxygen tolerance, cell wall architecture, and division kinetics, placing stringent demands on the applicability and transformation efficiency of genome editing tools. The current technological repertoire for intestinal microbiota engineering encompasses Clustered Regularly Interspaced Short Palindromic Repeats (CRISPR)–CRISPR-associated (Cas) proteins systems, base and prime editors, and eliminate phage-derived recombination-transposition platforms (Table 1). Among these, CRISPR-Cas systems have emerged as the predominant technical framework by virtue of their programmable nuclease activity.

#### 2.1.1. CRISPR-Cas Systems: Precise Targeted Cleavage and Adaptation to Gut Bacteria

The CRISPR-Cas system serves as an adaptive immune mechanism in prokaryotes, achieving precise cleavage of target DNA through the coordinated action of Cas effector proteins and gRNAs. The structural features of distinct Cas proteins determine their applicability in gut microbial genome editing [70,71]. Cas9 recognizes NGG-type protospacer adjacent motif (PAM) sequences and achieves editing efficiencies up to 100% in facultative anaerobic model organisms such as *E. coli* [72]. However, its application in obligate anaerobic gut commensals is limited by restriction–modification systems that constrain transformation efficiency [73], low host homologous recombination capacity leading to impaired repair of DNA double-strand breaks [74], and additional challenges, including PAM constraints [70], off-target effects, and toxicity from high-level expression [75], with efficiencies frequently falling below 30% in *Bacteroides* and *Clostridium* species [74]. Cas12a recognizes TTTV (V = A, C, or G) PAM sequences and requires only a single CRISPR RNA (crRNA) for target recognition [71,76,77], thereby simplifying multiplex editing design. Catalytically inactive dCpf1 combined with CRISPRi strategies enables efficient transcriptional repression in multiple *Clostridium* species to regulate short-chain fatty acid and bile acid metabolism [74], though this system similarly faces limitations of temperature sensitivity, PAM restrictions, and efficiency variations among different homologs [70,76]. Cas3 achieves large-scale deletions spanning tens of kilobases through unidirectional processive degradation, with efficiencies reaching 94% to 100% in excising antibiotic resistance gene clusters; yet, it presents risks of undefined deletion boundaries and potential cell lethality when targeting essential genes [78,79]. For editing complex gut microbial communities, the combined application of ET-seq and DART systems enables species- and locus-specific editing independent of culture-based methods, and has been successfully demonstrated in infant gut microbiota [80,81]. Overall, the success of gut microbial editing is constrained by multiple factors, namely restriction–modification systems [74,82], inefficient DNA repair pathways [82], species-specific differences in genetic element compatibility [74,83], and interspecies variability in editing efficiency [84]. Future efforts toward homology-independent CRISPR-RNA-guided integrase systems [71,82], ET-seq-based rapid identification of editable community members [80], machine-learning-driven directed evolution of Cas proteins [82], and the application of engineered functional strains for disease diagnosis and targeted therapeutics [85] represent critical avenues for overcoming existing bottlenecks.

#### 2.1.2. Base Editing and Prime Editing: Precision Modification Without Double-Strand Breaks

Slow-growing nonmodel gut commensals, such as *Faecalibacterium prausnitzii* and *Akkermansia muciniphila*, possess weak DNA repair capabilities and often cannot survive double-strand breaks introduced by CRISPR-Cas systems, as these bacteria lack robust nonhomologous end joining repair pathways [86,87]. Base editing and prime editing circumvent this problem by completely avoiding double-strand breaks; they achieve precise modifications through single-strand nicks alone, substantially improving cell survival [86,88]. Base editors fuse catalytically inactive Cas9 nickase with deaminases to accomplish transition point mutations, including cytosine-to-thymine and adenine-to-guanine conversions; this approach offers dual value for knocking out virulence factors and optimizing metabolic functions [86,89]. In *Bifidobacterium*, the cBEST system achieves editing efficiencies of merely 16.7% to 75% in wild-type strains, with transformants remaining scarce; only after knocking out the host restriction–modification system does efficiency reach 100% [90]. This finding reveals restriction–modification barriers as the critical bottleneck for expanding base editing into nonmodel gut bacteria. Regarding fidelity, Brödel et al. successfully performed adenine base editing in the mouse gut using engineered phage particles; next-generation sequencing showed on-target editing efficiencies of approximately 98%, and no off-target mutations were detected at candidate sites with seven base mismatches [91]. Base editing has attained 93% editing efficiency in the mouse gut with stable maintenance for 42 days [91]. However, its mutational scope remains limited to transitions only.

Prime editing systems fuse Cas9 nickase with reverse transcriptase; through the 3′ extension of prime editing guide RNA, all twelve types of base substitutions and small insertions or deletions become possible. No exogenous DNA donor is required, fundamentally eliminating the risk of horizontal gene transfer (HGT) [87,92]. However, prime editing efficiency remains severely constrained in nonmodel gut bacteria. In wild-type *Escherichia coli*, initial efficiency falls below 0.1%, far below the 45% to 90% observed in *Mycobacterium smegmatis* [87]. Genetic screens identified *SbcB*, *XseA*, and *ExoX* as critical host limiting factors; these three DNA exonucleases act in the 3′→5′ direction, and their combined knockout boosts efficiency approximately 100-fold [87]. Whole-genome sequencing further demonstrates that prime editing achieves extremely high fidelity in *E. coli* chromosomal engineering, with only a single off-target nucleotide substitution detected [92]. Nevertheless, applying prime editing to obligate anaerobic commensals such as *Faecalibacterium prausnitzii* presents major challenges, as optimizing prime editing guide RNA (pegRNA) design and achieving anaerobic adaptation remain unresolved [87]. Extending prime editing to such organisms requires drawing on the cBEST strategy; host restriction–modification systems must be engineered in parallel with exonuclease repair pathways [87,90]. Overall, base editing achieves efficient in vivo gut editing but remains limited to transition mutations; prime editing offers a broader mutation spectrum and better suits environments sensitive to HGT risks, but it suffers from low efficiency and requires host engineering [87,92]. Future work must integrate both approaches, systematically characterizing and removing species-specific barriers, including restriction–modification systems, exonuclease activity, and anaerobic adaptation, to extend these technologies from model gut bacteria to the entire gut microbiome [87,90].

#### 2.1.3. Phage-Derived Recombination and Transposon Systems: Synergistic Enhancement of Editing Efficiency

For bacteria with limited homologous recombination efficiency (e.g., *Pseudomonas* spp.), standalone CRISPR-Cas systems often fail to achieve optimal editing outcomes. Synergistic application of phage-derived recombination systems and transposon technologies offers an effective solution to this challenge. The λ-Red recombination system, derived from bacteriophage λ, facilitates homologous recombination repair through the action of recombination proteins. When coupled with CRISPR-Cas9, this system enhances editing efficiency in *E. coli* to over 95% for gene recoding events [93,94]. Conversely, transposon-encoded CRISPR-Cas systems enable RNA-guided, programmable chromosomal integration of exogenous genes without reliance on specific attachment sites. Combined with CRISPR technology, this approach achieves efficient, site-specific insertions across diverse bacteria [95,96]. For instance, the single-plasmid INTEGRATE system consolidates multiple functional components into a unified vector backbone, enabling marker-free DNA integration with efficiencies approaching 100% and supporting large-fragment insertions up to 10 kb [95]. Through multiplexed CRISPR arrays, this platform facilitates parallel insertions at three genomic loci simultaneously within a single cell. Integration with the Cre-LoxP recombination system further permits precise excision and reversible regulation of inserted sequences. Moreover, INTEGRATE demonstrates broad host applicability, functioning efficiently not only in various Gram-negative bacteria, including *E. coli*, *Klebsiella oxytoca*, and *Pseudomonas putida*, but also enabling species-specific, precision-targeted integration within complex gut microbiota environments [95]. The MetaEdit platform developed by Gelsinger et al. achieves culture-independent, precise, durable, and large-scale in vivo genome editing of specific target strains within complex native gut microbial communities. MetaEdit enables high-efficiency, high-specificity genomic integration in murine and human intestinal strains, successfully inserting metabolic pathways up to 7.5 kb into target sites. By engineering native *Bacteroides* with exogenous inulin utilization pathways, this platform achieves reversible and dynamic regulation of edited strain abundance through dietary intervention. Furthermore, it accomplished the inaugural genetic modification of segmented filamentous bacteria (SFB), which remain unculturable in isolation, directly within the host [97].

**Table 1:** Application Comparison of CRISPR-Cas Systems in Intestinal Bacteria.

Tool Name	Cas9 (SpCas9)	Cas12a (Cpf1)	Cas3 (Type I CRISPR)	Base Editing (CBE/ABE)	Prime Editing (PE)	CAST (CRISPR-Associated Transposase)
PAM Recognition	5′-NGG-3′	5′-TTTN-3′ (i.e., 5′-TTN-3′): FnCas12a, a naturally relaxed PAM	Type I-C: 5′-TTC-3′; Type I-B: 5′-CCW-3′; Type I-E: 5′-AAG-3′ (PAM sequence varies by Type I subtype; Cascade recognizes PAM via Cas8 subunit)	5′-NGG-3′ nCas9: D10A (CBE)/H840A (ABE)	5′-NGG-3′ (SpCas9 H840A nickase)	Type I-F: 5′-CC-3′ (canonical); 5′-CN-3′ (permissive); Type V-K: 5′-TTN-3′/5′-TTTV-3′ (V = A/C/G) (lower fidelity)
Representative Target Gut Strains	*E. coli* K-12, EcN; *Lactobacillus*: *L. reuteri*, *L. casei*, *L. acidophilus*, *L. gasseri*, *L. paracasei*; *Clostridium*: * C. tyrobutyricum*	*Bacteroidota*: *B. thetaiotaomicron*, *B. fragilis*, *B. ovatus*, *B. uniformis*; *P. vulgatus*; *Firmicutes*: *C. sporogenes*	*E. coli:* K-12, *E. coli* MG1655, EcN; *Clostridioides difficile*	*B. thetaiotaomicron* VPI 5482, *B. fragilis* NCTC 9343, *B. longum* NCIMB 8809, *B. adolescentis* DSM 20083, *B. pseudolongum*; *E. coli* MG1655, *E. coli* UTI89	*EcN*; *E. coli* MG1655	*E. coli* MG1655, *K. oxytoca*, *P. vulgata*, *B. thetaiotaomicron*, *B. uniformis*, *B. fragilis*, *B. stercoris*, SFB (*C. savagella*)
Core Advantages	Versatile (knockout, insertion, replacement); Compatible with most common gut bacteria; Near 100% efficiency in optimized HDR in *E. coli*; Clear PAM requirement, predictable targeting; Mature sgRNA design tools	Broad targeting: 5′-TTTV/TTN-3 PAM covers AT-rich regions; crRNA-only, no tracrRNA required; Autonomous crRNA processing; aTc-inducible markerless editing; High specificity, minimal off-target effects	Large fragment deletion (7–424 kb) at near-100% efficiency; Low off-target risk via PAM + crRNA dual recognition; Lower cytotoxicity than Cas9 (Type I-E in EcN)	No DSB, high cell viability; No HDR template required; Enables point mutations/premature stop codons; Multiplexed editing (≤4 genes) (pnCasBS-CBE); RM knockout dramatically improves efficiency(cBEST); Compatible with engineered phages for in vivo delivery(ABE8e/evoAPOBEC1-CBE)	No DSB, no donor template; 12 base conversions, small indels (≤97 bp del, ≤33 bp ins, efficiency drops sharply >10 bp); Low off-target (~15 vs. iSTOP ~71.5, XSTART ~52.3); ARG-free platform; Barcoding enabled	Large fragment insertion (>10 kb), no DSB; Site-specific integration (~48–50 bp from target); Low toxicity, multiplexed (3 loci), marker-free - ~100% on-target specificity (Tn-seq verified); gRNA-programmable across multiple species
Key Limitations	Limited efficiency in obligate anaerobes (RM system barrier, low HDR); complete vector typically >10 kb (promoter, selection marker, gRNA cassette); Cas9 toxicity in some *Clostridium* species; NGG PAM restricts target sites; ~26% unintended mutation frequency after dsDNA break repair in *E. coli*	Low HR repair efficiency in Gram-positive bacteria; Reduced FnCas12a activity in eukaryotic hosts; DSB-induced lethality in NHEJ-deficient bacteria; CRISPRi-dCpf1 vectors >10 kb reduce conjugation efficiency	Stochastic deletion boundaries; Complex multi-protein deliveryReduced efficiency near essential genes (2–25%); Insertion/replacement requires PEHR co-delivery	Only specific base conversions (C: G→T: A or A: T→G: C); Cannot insert large fragments; Editing window restricted (P4–P8/9); PAM (NGG) dependency; BE/sgRNA expression requires strain-specific optimization; RM barriers limit transformation efficiency	Complex pegRNA design (PBS + RT optimization); Efficiency drops with larger edits (>10 bp); Low in WT (<0.1–15%) without exonuc. Inhibition; 48 h induction for optimal eff; Obligate anaerobe optimization required	Requires TnsABC + TniQ-Cascade components; Narrow host range for some variants; Self-targeting inactivation, target immunity; Fitness cost from constitutive CAST expression; Type V-K: lower fidelity (~20–40% on-target)
Typical Scenarios	Routine gene insertion/replacement; *E. coli* high-throughput knockout screening; probiotic metabolic engineering; chassis strain development; edited strain barcode labeling	Gene deletion and insertion of intestinal *Bacteroidota*; Multiplex metabolic gene transcriptional suppression of intestinal Clostridia; In vivo regulation of gut microbiota; Manipulation of metabolic gene clusters	ARG cluster elimination (in vivo 80–97% blocking); Large virulence island removal (up to 424 kb); Pathogen-specific killing (*C. difficile*) Genome minimization and plasmid curing	Metabolic enzyme optimization; Virulence gene inactivation; Antibiotic resistance gene (*bla*, *aph*) inactivation; Probiotic metabolic engineering; Gut in situ precision editing; Microbiome-targeted therapeutics	Point mutations; Small indels; Codon modificaTion; Engineered Probiotics; Strain barcoding; ARG-free safe engineering	In situ gene therapy (intestinal microbiome); Stable implantation of large functional genes; Elimination of antibiotic resistance gene clusters; Metabolic pathway engineering (e.g., 7.5-kb PUL); Unculturable microbe editing (e.g., SFB); Diet-responsive strain control (e.g., inulin-Bt)
Editing Efficiency Range (%), [Representative Strain, Condition]	In vitro *E. coli* K-12/MG1655: ~65–82%; *Lactobacillus reuteri*: 90–100% via ssDNA recombineering; *Lactobacillus casei*: 25–62% using nCas9 nickase; *Lactobacillus acidophilus*, *L. gasseri*, *L. paracasei*: 35–100% via nCas9; *Clostridium tyrobutyricum*: 25% after removing RM system and endogenous plasmids; *Bacteroides thetaiotaomicron*: ~40%, lower than FnCas12a	In vitro *Bacteroides thetaiotaomicron*: gene deletion ~60%, GFP insertion >80%, 48 kb deletion ~6%, 5/10 kb deletion ~100%; *Prevotella vulgatus*: editing efficiency up to 100%; *Bacteroides fragilis*, *B. ovatus*, *B. uniformis:* editing efficiency >60%	In vitro *E. coli* MG1655: Editing efficiency for *lacZ*: 51–90%; for near-essential gene *hemB*: 2–25%; In vivo EcN (zebrafish gut): CRISPR-Cas3 inhibited the transfer of three antibiotic resistance genes (*mcr-1*, *NDM-1*, tet(X)) with efficiencies ranging from 80% to 97%.	In vitro pnCasBS-CBE: *Bacteroides thetaiotaomicron*: BT0793 100%, BT2804 80%, BT3788-1 70%, BT2158-1 80%, BT2802 66.7%, Non-model gut species: 66–100%; cBEST: Wild-type *Bifidobacterium longum*: cBEST2 ~27%, cBEST3 ~17%, cBEST4 ~75%; RM triple mutant: SpeE 100%, MetK 100%, bsh 100%; *Bifidobacterium adolescentis*: (Sau3AI) 100%; ABE8e: *E. coli* MG1655 > 99%; evoAPOBEC1-CBE: *E. coli* MG1655 > 99%, *E. coli* UTI89: *clbH* ~92%, *clbJ* ~83%, *cnf1* ~53%	In vitro EcN (48 h): substitution: 66.7%, insertion: 52.0%, deletion: 25.0%; Wild-type *E. coli* MG1655: deletion: ~26%, insertion: ~12.2%, substitution: ~6.8%; *E. coli* MG1655 Δ*sbcB*Δ*xseA*Δ*exoX*: up to ~40% editing efficiency at *xylB* locus	In vitro *E. coli* MG1655, BW25113: ~100%; *P. vulgatus*: >50–70% (pME0008); Multiplex: 3 loci simultaneously In vivo (murine gut): *P. vulgatus*: ~5% (day 3); *B. uniformis*: ~2%; *B. thetaiotaomicron*: ~1.5% peak, 99.8% specificity; SFB (C. savagella): ~2.3% (day 28), 100% specificity
References	[70,71,72,73,74,75,98,99,100,101,102]	[70,71,74,76,102,103,104,105]	[78,79,106,107,108]	[86,88,89,90,91]	[87,92,109]	[93,95,96,97]

### 2.2. Innovation of Delivery Systems

The extreme physicochemical conditions of the intestinal tract, encompassing gastric acid (pH 1.5–3.5), the detergent activity of bile acids, and continuous peristaltic motility, coupled with biofilm barriers and deep mucosal colonization characteristics of target organisms, collectively constitute formidable delivery barriers that compromise the structural integrity and functional activity of gene editing tools prior to reaching their intended targets. Contemporary strategies address these challenges through three primary vector systems (Table 2): engineered bacterial chassis that leverage the intrinsic colonization capacity of commensal organisms to achieve sustained in situ release; bacteriophage-based vectors that exploit strain-specific recognition mechanisms for precise targeting; and nanoparticle platforms that utilize advanced materials engineering to enhance environmental resilience and mucosal penetration capabilities.

#### 2.2.1. Phage Vectors: Precise Delivery with Host Specificity

As obligate bacterial viruses, bacteriophages represent ideal targeting delivery vehicles by virtue of their stringent host specificity; however, their application has been historically hampered by narrow host ranges and constrained genomic cargo capacities. The host range of a phage is primarily determined by tail fiber proteins located at the distal end of the phage tail, which mediate the initial reversible and specific recognition of susceptible bacteria through interaction with surface receptors such as lipopolysaccharide (LPS), outer membrane porins (e.g., OmpC), teichoic acids, and flagella [110]. At the molecular level, the receptor-binding domain at the tip of tail fibers engages in weak, dynamic interactions with bacterial surface receptors, enabling a “touch and search” mechanism that allows phages to randomly walk across the bacterial surface to locate optimal infection sites prior to irreversible attachment and genome injection [110]. Diversity-generating retroelements (DGRs) can further modify tail fiber proteins to alter host tropism, enabling individual temperate phages to infect a broad range of bacterial species, thus enhancing their adaptability within complex microbial communities [111]. To circumvent these limitations, researchers have employed directed evolution strategies involving mutational screening of tail fiber tip domains to successfully expand receptor recognition spectra [112]. Regarding cargo optimization, vectors constructed by inserting CRISPR-based systems into non-essential regions of λ phage have demonstrated versatile editing capabilities: dCas9-mediated transcriptional repression systems enable targeted gene silencing in *E. coli* while preserving cellular viability [102,113], whereas CRISPR-Cas3-equipped λ phage achieves sequence-specific antibacterial killing of pathogenic *E. coli* strains with enhanced efficiency [107]. More recently, engineered λ phage delivering DART (CRISPR-associated transposase) systems have accomplished kilobase-scale gene insertions and targeted disruptions with editing efficiencies exceeding 50% in monocultures and mixed bacterial communities [114], and phage-delivered cytosine base editors (CBEs) have enabled precise in situ point mutations within synthetic microbial communities [115]. The cardinal advantages of such platforms lie in their high targeting specificity and minimal off-target risk, with therapeutic potential having been extensively validated in animal models, exemplified by the engineered phage cocktail SNIPR001 featuring tail fiber engineering and a CRISPR-Cas payload, which has demonstrated efficient reduction of *E. coli* burden in mice [116]. Notably, studies utilizing M13 filamentous phage to deliver CRISPR-Cas9 systems have achieved strain-specific clearance and efficient genomic deletion of target *E. coli* strains within the murine gut, substantiating the efficacy of this strategy for precision editing of the intestinal microbiota in vivo [117]. It must be emphasized, however, that all successful in vivo demonstrations of phage-mediated CRISPR delivery reported to date have been limited to *E. coli*, a facultative anaerobe that serves as a convenient model organism. The extension of these strategies to obligate anaerobes, which comprise the majority of the human intestinal microbiota, remains a critical unmet challenge, as the technical barriers associated with phage propagation, infection dynamics, and CRISPR expression under strict anaerobic conditions have yet to be systematically overcome.

#### 2.2.2. Nanoparticle Vectors: Efficient Delivery Across the Mucus Layer

The intestinal mucus layer ranges from tens to hundreds of micrometers in thickness and consists of a dense mucin network, which effectively prevents exogenous gene editing cargoes from reaching targets within the intestinal lumen. Nanoparticles with diameters of 100 to 200 nm, by virtue of their appropriate size and surface modifiability, are regarded as ideal carriers for mucus penetration and subsequent microbiome editing. Current design strategies must address three hierarchical challenges: mucus penetration, targeted recognition, and intracellular delivery of CRISPR-Cas into target cells. Surface polyethylene glycol (PEG)ylation can minimize nonspecific mucin adsorption and enhance mucosal penetrability, as demonstrated by Lei et al., who showed that precisely PEG1500 shielded selenium nanovaccines not only achieve excellent mucus penetration but also efficiently activate mucosa-associated lymphoid tissues to induce local immune responses [118]. Peptide functionalized delivery systems can protect macromolecular cargoes from destruction under harsh intestinal conditions. Jing et al. employed HIV-derived TAT-cell-penetrating peptides to functionalize milk-derived extracellular vesicles, achieving 95.7% efficient GFP loading with 73.7% remaining intact after simulated gastrointestinal fluid treatment [119]. In the context of CRISPR-Cas nanoparticle delivery, Lin et al. constructed an oral micro- to nanogenome editing system (RNP NCs@dOMV@CAM) that employs calcium alginate microspheres as a gastric protective layer and utilizes probiotic-derived, lipopolysaccharide-depleted outer membrane vesicles (dOMVs) to traverse the colonic mucosal barrier and target inflammatory macrophages, achieving conditional Cas9 activation and TNF-α gene editing under inflammatory conditions and demonstrating the feasibility of oral nanoparticle delivery across intestinal barriers [120]. Kim et al. identified two candidate carriers, LNP 496 and LNP 470, containing the cationic lipid DOTAP through high-throughput screening of 511 lipid nanoparticle (LNP) formulations, which could deliver CRISPR-Cas13a plasmids into *Escherichia coli* with the assistance of polymyxin B. In a bacterial infection model, the LNP-Cas13a-gRNA treatment group improved mouse survival from 10% to 70% [121]. However, the study by Kim et al. employed intraperitoneal injection and has not yet achieved oral intestinal targeted delivery, relying on membrane disruptors to overcome the outer membrane barrier of Gram-negative bacteria. Meanwhile, although the system by Lin et al. successfully achieved oral intestinal delivery and host targeted gene editing, its target was inflammatory macrophages rather than intestinal commensal bacteria themselves. To date, no nanoparticle-mediated CRISPR-Cas system has successfully achieved targeted in vivo gene editing of intestinal commensal bacteria, particularly the dominant functionally core obligate anaerobes. Future research must urgently develop synthetic nanocarriers that simultaneously possess mucus penetration, obligate anaerobe targeting recognition, and autonomous bacterial cell wall crossing capabilities in order to bridge the gap from proof-of-concept to precise in vivo application of nanoparticle-mediated intestinal microbiome editing.

#### 2.2.3. Engineered Bacterial Vectors: Sustained Delivery via Live Colonization

Engineered bacterial chassis employ probiotics as programmable delivery platforms, harboring gene editing tools that enable sustained expression and in situ delivery following intestinal colonization. The core advantage of this strategy resides in the dual characteristics of intrinsic probiotic colonization capacity and synthetic biology regulation. Probiotic chassis inherently possess gastrointestinal colonization capabilities and establish immune tolerance with the host, while synthetic gene circuits can be designed to respond to specific intestinal microenvironmental cues such as localized hypoxia or particular metabolites, thereby achieving spatiotemporal control over editing tool expression and delivery [122,123]. Selection of an appropriate chassis requires careful consideration of colonization properties and genetic tractability. The non-pathogenic intestinal commensal *Escherichia coli* Nissle 1917 (EcN) achieves robust colonization in the murine gut [122,123], and engineered derivatives cured of cryptic plasmids via CRISPR-Cas9 editing exhibit plasmid stability over approximately 90 generations of in vitro cultivation [122]. Coupled with its well-established genetic toolbox, these attributes have established EcN as one of the most widely employed chassis organisms to date [123]. In contrast, *Bifidobacterium animalis* subsp. *lactis* BB-12 exhibits transient colonization in humans [124], yet displays potent metabolic activity during its intestinal residence. Through production of acetate and lactate, it facilitates cross-feeding interactions that enhance short-chain fatty acid synthesis by other community members, thereby significantly modulating microbiota architecture [124]. Regarding delivery strategies, engineered live bacteria has demonstrated substantial in vivo application potential. Although engineered EcN carrying a laboratory-evolved conjugative plasmid eB-TP114 achieved >99.9% elimination of targeted antibiotic-resistant *E. coli* in the mouse gut, this study relied on streptomycin pretreatment, used artificially introduced strains as targets, and the CRISPR-Cas9 system functioned as bacterial killing rather than precise genome editing [125]; by contrast, conjugative delivery of CRISPR-associated transposase systems via an engineered *E. coli* donor has achieved precise editing of *Bacteroides thetaiotaomicron* within the endogenous gut microbiota with >99% targeting specificity [97]. Furthermore, EcN engineered with a type I-E CRISPR-Cas3 system successfully blocked the horizontal transfer of multiple antibiotic resistance genes in the zebrafish gut [108]. Circuit design constitutes the cornerstone of precision control. The strictly anaerobic colonic environment activates anaerobic promoters such as *nirB* and *fdhF* to drive heterologous protein expression, whereas their activity is markedly reduced in the moderately hypoxic small intestinal milieu, potentially enabling spatial restriction of transgene expression to the colon [126,127]. Engineered FNR-dependent promoters have achieved more refined regulation, with anaerobic-activated variants demonstrating 24- to 138-fold enhanced activity under anoxic versus aerobic conditions and anaerobic-repressed variants dropping to 8–17% of baseline activity. These systems respond rapidly to oxygen depletion (within 30 min) and have been successfully implemented in proof-of-concept metabolic engineering applications, achieving pyruvate titers of 5.76 g/L at 55.7% yield [128], providing a theoretical foundation for precision intestinal therapeutics based on high-dynamic-range responses. Notably, oxygen sensing alone cannot fully discriminate between anatomically adjacent intestinal regions. Future iterations may incorporate multi-input logic gates combining bile-acid-responsive or pH-sensitive elements to further enhance targeting fidelity [126]. A critical consideration for clinical translation remains the immunogenicity of exogenous editing proteins such as Cas nucleases. This challenge can be mitigated through DNA-free editing systems involving ribonucleoprotein (RNP) delivery or inducible bacterial lysis strategies that minimize prolonged exposure to foreign antigens [126]. Careful chassis selection combined with circuit optimization will be essential for advancing engineered bacterial vectors from preclinical proof-of-concept to therapeutic reality.

**Table 2:** Comparison of Three Classes of Delivery Systems.

Delivery System Type	Bacteriophage Vectors	Nanoparticle Vectors (e.g., LNP, PBAE)	Engineered Bacterial Vectors (e.g., EcN)
Host Specificity	High (vast majority infect specific bacterial species/strains; some engineered phages exhibit broad host range)	No intrinsic host specificity; cell-type-dependent delivery efficiency; targeted delivery achievable through surface modification (targeting specific cells/tissues)	Variable (no strict host species limitations; colonization efficiency is microbiome-dependent)
Genetic Loading Capacity	Highly variable by type: T4 natural genome ~171 kb; T7 packaging limit <2.2 kb; λ genome ~48.5 kb, max effective payload insertion ~5 kb (without replacement), up to ~10.8 kb with replacement of non-essential regions (1–8.5 kb); M13 genome ~6.4 kb with expandable capsid	Relatively high (>95% encapsulation); challenging to achieve efficient encapsulation of large-size plasmids	Relatively high (plasmid or chromosomal integration; large DNA payload capacity)
Biocompatibility	High safety (well tolerated via oral delivery; no systemic exposure; does not disturb gut microbiota; validated in murine and minipig models)	Relatively high (toxicity can be reduced through material modification)	Extremely high (probiotic chassis; capable of intestinal colonization)
Core Advantages	Precise bacterial targeting with minimal off-target effects; host specificity can be tuned via tail fiber engineering	Penetrate mucus layers after surface modification; broad applicability (non-viral, no host range limitation)	Capable of in vivo colonization for sustained delivery; can be designed with environmentally responsive genetic circuits
Primary Challenges	Narrow host range (majority are narrow-host); prone to developing phage resistance; requires coating for oral delivery	Unmodified LNPs easily adsorbed and trapped by mucus; extremely low intestinal absorption efficiency (unmodified <5%; with bile acid modification: up to ~47%); reduced efficiency for large-size plasmids (9–19 kb); insufficient batch-to-batch consistency in manufacturing; uncontrollable size, surface charge and pDNA loading	Chassis colonization capacity affected by intestinal microbial competition; genetic stability requires optimization
References	[107,110,112,113,114,115,116]	[129,130,131,132,133]	[108,122,123,125,126,127]

### 2.3. Targeting and Controllability Design

To achieve precision spatiotemporal control over gut microbiome editing, current strategies employ a multi-layered safeguard system built upon two synergistic dimensions: species-specific recognition and environmentally responsive triggering [134]. At the species-specific level, researchers exploit the molecular incompatibility of target-exclusive promoters to strictly confine editing machinery activation within the intended taxon. For instance, the 16S rRNA promoter *pBT1* derived from *Bacteroides thetaiotaomicron* leverages its characteristic −7 and −33 consensus sequences to exclude heterologous transcriptional machinery from *E. coli* and other non-target species, thereby restricting editing activity exclusively to the desired lineage [135,136]. Building on this principle, Gelsinger et al. employed the *P1* promoter to drive guide RNA expression and, via a conjugative CAST delivery system, achieved efficient metagenome editing of *B. thetaiotaomicron* and related *Bacteroidaceae* commensals in the murine gut. This system not only demonstrated greater than 99% specificity in targeted tagging but also enabled integration of metabolic pathway payloads up to 7.5 kb in length. Through inulin utilization capability at the PUL locus, edited strains gained a selective fitness advantage upon dietary inulin supplementation, achieving 30- to 40-fold population enrichment [97]. In parallel, Huang et al. coupled the *P1* promoter with CRISPRi technology, utilizing an inducible *P1* promoter to regulate single guide RNA (sgRNA) expression and thereby realizing species-specific asymmetric adaptive regulation in a dual-species consortium [137]. To further mitigate off-target risks computationally, tools including CRISPRdirect, Cas-OFFinder, and CRISOT enable whole-genome prediction of potential off-target sites during the guide RNA design phase [138,139,140]; among these, CRISOT performs whole-genome off-target prediction based on molecular dynamics simulations of RNA–DNA interaction fingerprints. More recently, CCLMoff, an RNA language model-based approach integrating training data from thirteen off-target detection technologies, has demonstrated AUROC values of 0.985 in cross-dataset validation, exhibiting exceptional generalization capacity and predictive accuracy while effectively overcoming the limitations of earlier tools in predicting unseen sequences [141]. Complementing these in silico approaches, Kulcsar et al. established Cas9 fidelity cleavage rules and, based on these, constructed the CRISPRecise toolkit encompassing 19 high-fidelity SpCas9 variants. When paired with GUIDE-seq genome-wide off-target detection for target-matching screening, this platform drives off-target editing to undetectable levels, effectively resolving the longstanding trade-off between efficiency and specificity [142].

On the environmental response front, site-specific strategies harness the intestinal pH gradient to achieve segment-specific regulation. The acid-responsive promoter pAsr, for example, is strongly induced in *E. coli* under external acidic conditions (pH < 5.0), with peak activity at approximately pH 4.0 to 4.5, providing a theoretical molecular basis for segment-specific transcriptional control in the gut, though direct translation to in vivo intestinal editing remains experimentally unvalidated [143,144]. A more mature approach employs pH-sensitive materials and chemical engineering to co-encapsulate engineered bacteria; Jiang and colleagues used polyelectrolyte complex coatings to modify EcN, enhancing engineered bacterial survival in the gastrointestinal tract by 40- to 74-fold, while simultaneously leveraging genetic editing to overexpress the siderophore microcin, which targets and eliminates pathogenic bacteria through a “Trojan horse” mechanism; this synergistic strategy achieved both anti-inflammatory efficacy and microbiome modulation in a model of UC, offering a new paradigm for stable delivery and precise release of live bacterial editing tools [145]. Pathology-responsive strategies further endow editing systems with intelligent intervention capability, enabling them to sense disease-specific signals and generate therapeutic outputs at lesion sites. Engineered EcN carrying a nitric-oxide-responsive genetic circuit, for instance, can detect elevated NO concentrations at colonic inflammatory sites and trigger local expression of anti-TNF-alpha nanobodies to neutralize pro-inflammatory cytokines [146]. Complementing this strategy, Luo et al. developed M2 macrophage membrane-coated nanorobots encapsulated in alginate microspheres that, leveraging membrane surface receptors for specific recognition of inflammatory macrophages and intestinal epithelial cells, accumulate in inflamed tissue and neutralize inflammatory factors through receptor–ligand interactions while simultaneously driving M1-to-M2 macrophage repolarization, thereby exerting potent anti-colitis therapeutic effects [147]. Collectively, these hierarchical designs spanning molecular recognition, material responsiveness, and cell-mimetic delivery constitute a paradigm shift from passive intervention to active adaptation in precision microbiome editing.

## 3. Therapeutic Applications and Case Studies

Gut microbiome engineering offers a novel therapeutic paradigm for intractable conditions, including metabolic disorders, IBD, cancer immunotherapy, and neuropsychiatric diseases, through precision manipulation of microbial genetic circuits or metabolic networks. While current investigations remain predominantly at the stage of ex vivo proof-of-concept validation and preclinical animal modeling, with limited accumulation of systematic clinical efficacy data, synthetic biology design strategies, encompassing the construction of environmentally responsive genetic circuits, targeted biosynthesis of therapeutic molecules, and modulation of microbe–host interactions, have demonstrated theoretical potential to remodel gut microecological architecture and achieve durable precision interventions. The following sections delineate disease-specific microbiome editing strategies and their applications.

### 3.1. Metabolic Diseases: Modulation of Energy Homeostasis and Glycolipid Metabolism

One of the core pathological mechanisms underlying metabolic disorders, including obesity, type 2 diabetes (T2D), and non-alcoholic fatty liver disease (NAFLD), involves the structural imbalance of gut microbiota metabolic function. This dysregulation is characterized by decreased abundance of SCFA-producing taxa and increased lipopolysaccharide (LPS) translocation. Targeting these pathological foundations, microbiome engineering is opening new avenues for the treatment of metabolic diseases through the directed modification of strain metabolic capabilities or stress resistance.

In the field of microbiome engineering, proof-of-concept studies that target metabolic diseases through directed modification of bacterial metabolism or stress resistance have achieved major breakthroughs. These investigations span not only obesity and T2D but also complex multi-organ metabolic disorders of the gut–liver–brain axis. Russell et al. isolated a native *Escherichia coli* strain, EcAZ-1, from feces of conventionally raised mice and engineered it to express functional genes including bile salt hydrolase (BSH), creating an optimized chassis for live bacterial therapeutics. This engineered strain achieved permanent engraftment in the host gut without antibiotic pretreatment, with persistence reaching statistical significance at *p* < 0.0001. In the Ob/Ob genetic model of diabetes, a single administration enhanced host insulin sensitivity at *p* < 0.05 and lowered postprandial blood glucose over 15 weeks, thereby reversing diabetic pathophysiology in a sustained manner [148]. In parallel, Mao et al. subjected probiotic *E. coli* Nissle 1917 to directed evolution under iterative hydrogen peroxide stress and isolated the engineered strain REcN with enhanced tolerance to reactive oxygen species. Surface functionalization with fructooligosaccharide–calcium carbonate composites yielded REcN-F/Ca. In high-fat-diet-induced obese male C57BL/6J mice, this engineered probiotic activated PPAR signaling pathways, enriched butyrate-producing taxa such as Lachnospiraceae and Blautia, and restored intestinal short-chain fatty acid levels at *p* < 0.0001. These effects translated into a 25.4% reduction in weight gain, markedly improved glucose homeostasis, and a 73.2% decrease in the homeostatic model assessment for insulin resistance (HOMA-IR), all at *p* < 0.0001 [149]. Furthermore, Oh et al. constructed an engineered *Lactobacillus reuteri* strain that stably secretes recombinant interleukin-22. In a diet-induced obesity mouse model, eight weeks of treatment significantly reduced both liver weight and hepatic triglyceride levels at *p* < 0.001, exceeding the baseline improvements seen with wild-type probiotic therapy and providing proof-of-concept for live bacterial therapeutics in non-alcoholic fatty liver disease [150]. Extending this paradigm to multi-metabolite disorders of the gut–liver–brain axis, Aggarwal et al. engineered two *Lactobacillus plantarum* strains, Lp-NH3 and Lp-Q, to simultaneously achieve ammonia consumption, branched-chain amino acid (BCAA) synthesis, and L-glutamine metabolism. Lp-NH3 couples ammonia assimilation with BCAA biosynthesis, while Lp-Q enhances glutamine utilization to block ammonia regeneration. In two preclinical hepatic encephalopathy models (hyperammonemia and bile duct ligation models), the Lp-NH3^+^Q cocktail reduced serum and brain ammonia concentrations by up to 10-fold, restored BCAA and glutamine balance, and significantly improved anxiety-like behavior and cognitive function. Brain transcriptomic analysis further revealed restoration of neuronal signaling and attenuation of neuroinflammation. This engineered cocktail outperformed rifaximin in therapeutic efficacy while preserving gut microbiota diversity; the strains were completely cleared within 72 h after dosing cessation with a favorable safety profile [151]. Collectively, these studies demonstrate that microbiome engineering offers broad therapeutic potential against metabolic diseases through precision functional modification of bacterial strains. The approaches span directed insertion of functional genes into native chassis strains, adaptive evolution coupled with surface engineering to enhance stress resistance, engineered secretion and delivery of therapeutic proteins, and multi-metabolite pathway rewiring for simultaneous modulation of interconnected metabolites across the gut–liver–brain axis.

### 3.2. IBD: Mucosal Barrier Restoration and Inflammation Attenuation

The core pathological mechanisms of IBD involve disruption of the intestinal mucosal barrier and immune-mediated chronic inflammation, with gut microbiota dysbiosis serving as a critical driver characterized by the overexpansion of adherent-invasive *E. coli* (AIEC) and depletion of butyrate-producing commensals. Microbiome engineering achieves precise intervention through a dual strategy encompassing pathogen virulence inactivation and enhancement of local anti-inflammatory capacity [152]. The former employs CRISPR-Cas systems to neutralize AIEC virulence genes in situ or inhibit their colonization, while the latter utilizes engineered bacteria to locally secrete anti-inflammatory molecules such as IL-10 and anti-TNF-α nanobodies to remodel the immune microenvironment [152,153]. Engineered bacteria-mediated local delivery of anti-inflammatory metabolites provides sustained support for mucosal repair. Gong et al. introduced a heterologous butyrate biosynthesis pathway into EcN, achieving in situ butyrate delivery at 1.4 g/L in artificial intestinal environments; this strain significantly ameliorated dextran sulfate sodium (DSS)-induced chronic colitis, restored mucosal barrier integrity, and predominantly depended on GSDMD–HDAC3 pathway inhibition, wherein butyrate functions as an HDAC3 inhibitor to suppress gasdermin D-mediated pyroptosis [154]. Additionally, EcN has been engineered to synthesize the ketone body (R)-3-hydroxybutyrate (3HB), enabling sustainable production within the intestinal lumen and alleviating DSS-induced colitis [155].

Significant progress has been achieved in engineering probiotic bacteria for targeted anti-inflammatory therapy. A dual-engineered EcN system comprising EcN-TNFαNb and EcN-IL10, respectively expressing anti-TNF-α nanobodies and IL-10, significantly ameliorated DSS-induced colitis in mice while reducing inflammatory cell infiltration and pro-inflammatory cytokine levels [153]. Hua et al. constructed EcNΔ*lpp*::*A5-aTN* displaying ANXA5 to target inflammatory sites and secrete anti-TNF-α nanobodies, outperforming wild-type EcN and infliximab in DSS-induced UC by suppressing reactive oxygen species (ROS)/JNK/p38/Caspase-mediated intestinal epithelial cell apoptosis [141]. The PATCH system employed engineered EcN to secrete curli fibers displaying trefoil factors (TFFs), where CsgA-TFF3 fusion proteins demonstrated protective effects by lowering IL-6, IL-17A, and TNF-α levels, enhancing mucosal barrier function, and reducing Th17 responses [156]. Furthermore, smart responsive engineered probiotics represent a new frontier, as Weibel et al. engineered EcN with an NO biosensor (NorR-pNorVβ) that senses the inflammatory biomarker NO and utilizes the type I hemolysin A secretion system to secrete anti-TNFα nanobodies with binding affinity comparable to adalimumab [146].

Beyond anti-inflammatory molecule delivery, advanced engineering strategies have explored antioxidant protection and targeted pathogen elimination. Cao et al. developed artificial enzyme-armed *Bifidobacterium longum* (BL@B-SA50) using single-atom catalyst Fe SA to mimic SOD and CAT antioxidant enzymes, effectively scavenging ROS and demonstrating therapeutic potential in mouse UC and CD models as well as beagle dog colitis [157]. Guo et al. engineered *Lactobacillus casei* with embedded selenium nanodots (Se-fLac), where the periplasmic film enhanced gastric acid resistance and mucosal adhesion while selenium nanodots cleared ROS, alleviating inflammation in multiple mouse models and non-human primates [158]. Jiang et al. applied a polyelectrolyte complex coating to EcN that improved gastric survival by 40-fold and small intestine survival by 74-fold, while the EcN::*mcmA* strain overexpressed the siderophore microcin MccM, exploiting a “Trojan horse” mechanism to target and destroy pathogenic bacteria in DSS-induced UC models [145]. Collectively, these CRISPR-Cas9-mediated gene editing advances, encompassing targeted anti-inflammatory delivery, ROS clearance, barrier restoration, and microbiota homeostasis, herald a new era of engineered probiotics for IBD therapy, with future intelligent systems capable of real-time inflammation monitoring and dynamic therapeutic release promising to further enhance clinical precision.

### 3.3. Cancer Immunotherapy: Remodeling the Tumor Microenvironment and Potentiating Anti-Tumor Immunity

The gut microbiota profoundly modulates tumor immunotherapy responses through the microbiota–metabolite–immune axis; its metabolites not only regulate dendritic cell cross-presentation and subsequent CD8^+^ T-cell-mediated anti-tumor effector functions but also exert direct anti-tumor effects. For instance, Yue et al. engineered *Escherichia coli* to produce antigen-bearing OMVs in situ within the intestine; these vesicles traverse the intestinal epithelial barrier and are taken up by lamina propria dendritic cells to mediate antigen presentation, eliciting robust anti-tumor immunity against melanoma and colorectal cancer [159]. Building upon this, they further developed an oral probiotic vaccine platform integrating dual-antigen ferritin nanoparticle scaffolds with a controllable bacterial lysis system; via RGD peptide-mediated M-cell-targeted delivery, this platform promotes mucosal dendritic cell antigen uptake and presentation, achieving durable protection in melanoma subcutaneous tumor and lung metastasis models [160].

At the spatiotemporal precision level, Chen et al. developed a lactate/NIR-responsive system that enables engineered bacteria to express and accumulate cytolysin A (ClyA) cytolysin within tumors and achieve precision lysis via near-infrared-triggered temperature-controlled circuits [161]. Zou et al. employed a lactate-sensing circuit coupled with the essential gene *asd* to confine EcN to tumor sites; quorum sensing systems then drive on-demand alpha-hemolysin release to kill tumor cells, while coagulase-mediated vascular thrombosis cuts off tumor blood supply and establishes a physical barrier to contain bacterial spread [162]. Complementarily, Xu et al. reconstructed the arginine–nitric oxide (NO) metabolic circuit to sustain NO synthesis, normalizing tumor vasculature, alleviating hypoxia, enhancing immune cell infiltration, and reversing T cell exhaustion [163]. Collectively, these strategies achieve spatially selective activation by exploiting tumor microenvironmental hallmarks, circumventing the off-target toxicities associated with systemic drug administration, and providing safe and feasible new paradigms for engineered bacteria-mediated precision tumor therapy.

### 3.4. Neuropsychiatric Disorders: Modulation of the MGB Axis

The pathogenesis and progression of neuropsychiatric disorders are intimately associated with dysfunction of the MGB axis. Lee et al. systematically elucidated the mechanisms by which gut microbiota modulate central nervous system function through multiple pathways encompassing neurotransmitter precursor metabolism, vagal afferent signaling, and microbial metabolite-mediated immune modulation, thereby establishing a theoretical foundation for microbiome engineering in neuropsychiatric therapeutics [164]. In the dopaminergic domain, Padhi et al. constructed a genetically engineered EcN system (EcN_L-DOPA) wherein the hpaBC operon was chromosomally integrated under the control of a programmable L-rhamnose-inducible promoter, enabling continuous, titratable biosynthesis of L-DOPA from dietary L-tyrosine; oral administration of EcN_L-DOPA combined with a peripheral decarboxylase inhibitor maintained stable therapeutic plasma L-DOPA concentrations, increased striatal dopamine levels, and notably alleviated depressive-like behaviors in murine and canine models without the pulsatile fluctuations associated with conventional oral L-DOPA therapy [165]. In the GABAergic domain, Wang et al. developed an alternating magnetic field (AMF)-responsive engineered probiotic system (EcN-GadABC@Fe3O4-NE) by transforming EcN with a temperature-sensitive plasmid expressing *gadA*, *gadB*, and *gadC* under the control of a thermal-responsive promoter; Fe3O4 nanoparticles coated on the bacterial membrane converted AMF stimuli into thermal signals, achieving non-invasive, spatiotemporally precise control of GABA release in the gastrointestinal tract, and oral administration of this system significantly attenuated anxiety-like behaviors in restraint-stressed mice through synergistic inactivation of the nucleus of the solitary tract and locus coeruleus via gastrointestinal vagal afferent signaling while concurrently restoring gut microbiota homeostasis [166]. Lebovich et al. further leveraged synthetic biology tools to implement hierarchical negative feedback gene circuits for precise dynamic regulation of GABA biosynthesis, constructing intelligent engineered probiotics capable of autonomously sensing GABA concentrations and regulating glutamate decarboxylase expression, thereby providing a programmable solution for maintaining gut–central GABA signaling homeostasis [167]. Needham et al. revealed from a microbial metabolite perspective that 4-ethylphenol (4EP) produced by bioengineered gut bacteria is sulfated by the host into 4-ethylphenyl sulfate (4EPS). This metabolite enters the brain and alters region-specific activity and functional connectivity across limbic circuits by impairing oligodendrocyte maturation and reducing axonal myelination, which induces anxiety-like behaviors in mice. Pharmacological promotion of oligodendrocyte differentiation reversed these myelination defects and prevented the behavioral abnormalities, providing causal mechanistic evidence for intervening in neuropsychiatric disorders through targeted modulation of specific microbial metabolites [168]. Collectively, these strategies spanning continuous dopamine precursor delivery, remotely controlled GABA release, autonomous feedback-regulated neurotransmitter synthesis, microbial metabolite-directed brain function modulation, and precision treatment systems ranging from microbiota transplantation to engineered commensal microorganisms demonstrate substantial potential for intervening in neuropsychiatric disorders through precise, multimodal manipulation of gut–brain axis metabolic signals by engineered probiotics, while underscoring the need for further translational studies to validate their clinical efficacy and long-term safety.

## 4. Challenges and Solutions

The clinical translation of gut microbiome engineering faces dual bottlenecks in editing efficiency and delivery precision. At the gene editing tool level, CRISPR-Cas systems are constrained by the restriction–modification systems of bacterial hosts [90]; the risk of HGT [169] and off-target effects [170] further limit their application in live bacteria editing. At the delivery system level, the dense mucin network of the intestinal mucus layer can trap phage vectors and other carriers, obstructing effective contact with target bacteria. Immunoglobulin-like domains on the phage capsid surface bind to mucin glycans, enriching phages in mucus by 4.4-fold while reducing their diffusion rate by 8-fold [171]. More critically, immune clearance constitutes the core obstacle to delivery: systemically administered phages face complement activation, opsonization, and reticuloendothelial system phagocytosis and can be cleared within minutes. PEGylation shields capsid antigenic epitopes through a hydrophilic steric barrier, significantly reducing anti-phage IgG and increasing the area under the curve by up to 54-fold [172].

Beyond delivery efficiency, the colonization stability of engineered bacteria following genetic manipulation represents another critical limiting factor. Due to ecological niche competition and the abundance of mobile genetic elements in the gut, engineered bacteria face the risk of clearance by wild-type microbiota, with HGT frequencies 10 to 100 times higher than in other environments [169]. In response, synthetic biology has provided innovative solutions through the establishment of nutritional complementarity. Degnan et al. revealed that over 80% of gut bacteria depend on exogenous corrinoids (represented by vitamin B_12_, yet fewer than 25% possess the capacity for biosynthesis. This metabolic supply-demand imbalance constitutes a natural cross-feeding network, providing an ecological foundation for engineered bacteria to achieve long-term colonization through metabolic reciprocity [173].

In addition to these technical and ecological challenges, clinical translation must also carefully address HGT as a biosafety risk. Engineered genetic elements can spread through three pathways: plasmid conjugation, mediated by pili for direct transfer between bacteria, occurs at frequencies 10 to 100 times higher in the gut than in other environments [169]; natural transformation occurs through uptake of free DNA from the environment; and phage transduction is mediated by lysogenic-to-lytic switching of temperate phages [169]. Phage transduction encompasses multiple mechanisms: specialized transduction arises from imprecise prophage excision (approximately 1/10^4^ lambda lambda phage particles) [174]; generalized transduction results from terminase misrecognition of host DNA (approximately 1.8% of P22 phage particles and 4.5% of P1 phage particles contain host DNA) [174]; and lateral transduction sequentially packages up to 7 headfuls (>300 kb) of host DNA at frequencies 2 to 3 orders of magnitude higher than generalized transduction [175,176]. Metagenomic evidence further demonstrates that 8.6% of mouse gut bacterial contigs carry transduction signals [174]. To address this risk, a multi-layered collaborative containment system has been established: chromosomal integration technology can reduce plasmid-mediated HGT frequency from 1.2 × 10^−5^ to less than <1 × 10^−7^ [169] events per cell [169]; an environmentally responsive kill switch achieves 99.9% mortality of engineered bacteria within 6 h at 39 °C, enabling self-destruction upon environmental escape [169]; and CRISPRi targeting *rbsB* gene silencing reduces conjugation transfer rates by 88.7% and biofilm formation by 68.2% [177]. The coordinated deployment of these strategies provides systematic safeguards for the safe clinical translation of gut microbiome engineering.

## 5. Conclusions and Future Perspectives

Gut microbiome engineering is undergoing a paradigm shift from proof-of-concept to clinical approval in specific indications, such as recurrent *Clostridioides difficile* infection [178], while continuing to advance across a broader disease spectrum. This synergistic approach overcomes the coarse intervention model of traditional microbial therapeutics, demonstrating the potential for precise reprogramming of gut microbiome structure and function in model bacterial strains and showing differentiated therapeutic advantages in metabolic diseases, IBD, and tumor immunomodulation alongside preliminary exploratory efficacy signals in neuropsychiatric conditions. The frontier of technological innovation centers on spatially specific editing and rational design powered by artificial intelligence. Engineered probiotics encapsulated in protective hydrogels and nanocarriers equipped with CRISPR in early exploratory stages serve as the technical foundation, complemented by large scale mining and utilization of endogenous CRISPR systems, progressively enabling in situ editing of model strains independent of ex vivo cultivation while expanding editing targets toward uncultured microorganisms that constitute the majority of species diversity. Meanwhile, the integration of multiomics data and deep learning is reshaping the design paradigm for editing strategies, leveraging neural network models to infer regulatory relationships in microbe–host–metabolic interactions and systematically optimizing guide RNA sequences, delivery vehicle formulation and component design, and gene circuit parameters in combination with traditional optimization algorithms, collaboratively enhancing editing efficiency and spatial specificity. Nevertheless, this pathway remains subject to multiple practical constraints. The high dimensional heterogeneity and batch effects of microbiome data introduce systematic bias into model training; the black box nature of deep learning undermines the interpretability of editing decisions; and the geographic and ethnic biases inherent in existing reference databases limit the cross-population generalizability of models. The scarcity of high-quality annotated data, the high costs of iterative experimental validation and computational prediction loops, and biosafety and ecological ethics considerations collectively constitute barriers that AI-driven strategies must overcome in transitioning from algorithmic optimization to clinical deployment. Clinical translation will exhibit dual characteristics of stratified medicine and multimodal synergy. Precision stratification based on patient microbiome heterogeneity with research stage AUC values of 0.89 to 0.95, alongside dynamic monitoring technologies currently in early-stage validation, is poised to drive therapeutic strategies from standardized regimens toward individualized adaptation, gradually realizing microbiome-guided precision intervention. Gut microbiome engineering will also form synergistic networks with immune checkpoint inhibitors, small molecule drugs, and nutritional interventions, breaking through the efficacy bottlenecks of monotherapy by modulating tumor microenvironment immune infiltration, optimizing host-drug-metabolism kinetics, or enhancing intestinal barrier function. As regulatory science systems undergo progressive refinement and manufacturing standards for live biotherapeutic products (LBPs) are progressively established, particularly regarding quality control frameworks, long-term safety evaluation guidelines under development, and the ongoing exploration and progressive maturation of ecological risk assessment frameworks, gut microbiome engineering is expected to complete the transition from experimental therapy to routine clinical practice in specific indication areas and progressively expand toward a broader disease spectrum, emerging as a microbial targeted therapeutic modality with substantial potential within precision medicine systems.

## Data Availability

No new data were created or analyzed in this study. Data sharing is not applicable to this article.

## Abbreviations

The following abbreviations are used in this manuscript:

IBD	Inflammatory bowel disease
FMT	Fecal microbiota transplantation
EcN	*Escherichia coli* Nissle 1917
HOMA-IR	Homeostatic model assessment for insulin resistance
SCFA	Short-chain fatty acid
GABA	Gamma-aminobutyric acid
gRNA	Guide RNAs
LPS	Lipopolysaccharide
BSH	Bile salt hydrolase
3HB	(R)-3-hydroxybutyrate
DSS	Dextran sulfate sodium
HDAC	Histone deacetylase
EPS	Exopolysaccharides
MGB	Microbiota–gut–brain
Treg	Regulatory T cell
HGT	Horizontal gene transfer
CRISPRi	CRISPR interference
LBP	Live Biotherapeutic Product
UC	Ulcerative colitis
CD	Crohn’s disease
AIEC	Adherent-invasive *Escherichia coli*
ASD	Autism spectrum disorder
PAM	Protospacer adjacent motif
pegRNA	Prime editing guide RNA
SFB	*S**egmented filamentous bacteria*
PEG	Polyethylene glycol
RNP	Ribonucleoprotein
CAST	CRISPR-associated transposase
T2D	Type 2 diabetes
NAFLD	Non-alcoholic fatty liver disease
H_2_S	Hydrogen sulfide
CNS	Central nervous system
BBB	Blood-brain barrier
ERK1/2	Extracellular signal-regulated kinase 1/2
MAPK	Mitogen-activated protein kinase
DGR	Diversity-generating retroelement
dOMV	Depleted outer membrane vesicle
LNP	Lipid nanoparticle
AMF	Alternating magnetic field
4EP	4-ethylphenol
4EPS	4-ethylphenyl sulfate
rCDI	Recurrent *Clostridioides difficile* infection
ROS	Reactive oxygen species
TNF-α	Tumor necrosis factor-α
NF-κB	Nuclear factor kappa-B
NO	Nitric oxide
BCAA	Branched-chain amino acid
OMV	Outer membrane vesicle
dCas9	Catalytically dead Cas9
sgRNA	Single guide RNA
GPR41	G protein-coupled receptor 41
IL-6	Interleukin-6
TLR2	Toll-like receptor 2
TLR4	Toll-like receptor 4
GPR43	G protein-coupled receptor 43
CBE	Cytosine base editor
ClyA	Cytolysin A
5-HT	5-hydroxytryptamine
AI	Artificial intelligence
CRISPR	Clustered Regularly Interspaced Short Palindromic Repeats
Cas	CRISPR-associated
FFAR	Free fatty acid receptor
